# Improving Clinical Handover on an Electronic Communication Platform: A Quality Improvement Project

**DOI:** 10.7759/cureus.19156

**Published:** 2021-10-31

**Authors:** Xiang Li Tan, Hyun Park, Jasmine Patel, Matthew Murden

**Affiliations:** 1 Medicine, Ashford and St. Peter's Hospital NHS Foundation Trust, Chertsey, GBR

**Keywords:** handover, continuity, patient care, sbar, covid-19

## Abstract

Background

National guidance stipulates the essential components of a safe handover. Shift-based work and the COVID-19 pandemic has led to an increased turnover and re-deployment of staff into new clinical areas, creating challenges in delivering effective handovers.

Aim

The aim of this quality improvement project (QIP) was to improve adherence to a local standardised handover proforma to improve the quality and consistency of handovers.

Methods

Handovers were assessed by measuring the completion rates of the essential components of a safe handover as outlined in the national guidance. Data were collected from an electronic handover system which follows the Situation, Background, Assessment and Recommendations (SBAR) structure, and percentage completion rates obtained for each component assessed. Following baseline measurement, four Plan-Do-Study-Act (PDSA) cycles were completed between August 2020 and February 2021 across two junior doctor rotations and during a COVID surge rota.

Results

A total of 710 handovers were assessed across the four PDSA cycles. There were overall improvements in the percentage completion rates of each component compared to baseline: Under ‘Situation’, admission dates increased by 13.7%, estimated discharge date by 33.3% and 100% completion rate maintained for the presenting complaint. Under ‘Background’, past medical history remained static, with a 12.1% increase in documentation of a social history. Under ‘Assessment’, escalation status increased by 335%, issues list by 242% and important updates by 35.2%. Under ‘Recommendations’, completion rate for plans was maintained at 100%.

Conclusions

Our findings demonstrated an overall improvement in the majority of components of the handover proforma. Challenges remain with the rotation of junior doctors through different specialties leading to a loss of institutional knowledge and reduced longevity of the intervention’s effect, exacerbated by the introduction of the COVID surge rota. A long-lasting improvement may require a shift to a completely electronic patient records system (ePR) which incorporates a handover tool.

## Introduction

COVID-19 is one of the greatest challenges faced by the National Health Service (NHS) with significant implications for clinician-to-clinician handovers of patient information. Large numbers of staff were re-deployed throughout the NHS with changes in shift patterns, including the introduction of COVID surge rotas. Furthermore, the implementation of reverse-transcriptase polymerase chain reaction (RT-PCR) as a primary diagnostic and triaging tool contributed to an increase in the frequency of patient transfers between wards based on their COVID-19 status, often without direct handovers between the clinicians. The increasing workload and staffing pressures caused by re-deployment, shielding, isolation and sickness, resulted in increased time pressures, affecting the quality of handovers at a time when good handovers are more important than ever.

Handover is an essential component of good patient care and ensures continuity of care. It describes the process of transferring the responsibility for immediate and ongoing care of patients between healthcare professionals [1]. It has been reported that the performance of handover varies significantly across the NHS [2]. The importance of handover is widely recognised and despite published frameworks provided by the Royal College of Physicians [3], British Medical Association (BMA) [4], Royal College of Surgeons (RCS) [5] and inclusion in the Foundation Programme curriculum for junior doctors (UKFPO) [6], there has been limited translation locally into improvements of handovers in clinical practice.

The quality of clinical handover has been correlated with the incidence of preventable cases of mortality and morbidity, particularly during shifts with reduced staffing levels [7]. The introduction of the European Working Time Directive (EWTD) and increasing specialisation has necessitated an increased frequency of handovers between healthcare professionals with an increased emphasis on team-working and a trend towards shift-based working [8]. In some medical specialities, such as intensive care and neonatal medicine, active handover is part of a well-established routine [2]. However, these infrastructure changes have not been met with adequate levels of training and education in other specialities. Furthermore, the ongoing COVID-19 pandemic has placed unprecedented pressures on the traditional healthcare model highlighting the critical need for continued optimisation of communication methods [9].

In recent years, advocacy for the implementation of electronic tools to facilitate handovers has increased [10]. At Ashford and St. Peter’s Hospitals NHS Foundation Trust (ASPH), Careflow Connect© was introduced in May 2017 through a staggered approach. Careflow handover consists of four subsections following the Situation, Background, Assessment and Recommendation (SBAR) structure, with free text boxes for each section, allowing handovers to be updated in real time. The system was firstly adopted by the surgical teams before becoming used Trust-wide in 2018. Currently, it is an integral aspect of the ward-based multidisciplinary team (MDT) handover process. It’s updated at daily ward rounds, during on-call handovers, and through referrals to some specialty services. Careflow is accessible to all members of the multidisciplinary team (MDT) including doctors, nurses, and allied health professionals. However, the structure of entries into the free text boxes on the Careflow handover frame is not standardised, and the quality of the handovers varies across the hospital. To address these issues, a quality improvement project (QIP) was undertaken at St. Peter’s Hospital. Our aim was to improve adherence to a standardised handover structure following the national frameworks over a six-month period across the hospital.

## Materials and methods

A pro forma which includes essential components as part of the SBAR handover recommended in the national guidance, such as the RCP Acute Care Toolkit, was used to assess the standards of handover (Table 1) [1]. In addition, we included ‘Issues’ as a required component, as this headline provides a snapshot of the current issues a patient is being treated for and opportunity to include any other significant information relevant to the clinical picture. Given that excessive clinical information may hinder effective handover [10], our accepted standard in the ‘Recommendation’ section was limited to three days’ worth of plans. To achieve our aim of improving the handover across the hospital, data was collected through the weekend handover lists, which includes a selection of patients from across the hospital, who require a clinical review over the weekend. We also collected data from three individual wards, randomly selected using a random number generator, across the four PDSA cycles. Outcome measures were a percentage completion rate for each component listed below:

**Table 1:** Handover proforma

Situation	Background	Assessment	Recommendation
Admission date (ADM)	Past medical history (PMH)	Escalation status and ceiling of care (ReSPECT)	Plans
Estimated discharge date (EDD)	Social history (SH)	Issues	
Presenting complaint (PC)			

● Situation: Admission date (ADM), Estimated discharge date (EDD), Presenting complaint (PC)

● Background: Past medical history (PMH), Social history (SH)

● Assessment: Escalation status, Issues, Important updates

● Recommendation: Plans including dates

Baseline measurements

Our baseline audit highlighted several key areas for improvement, with ADM (51%), EDD (60%), escalation status (20%) and issues (12%) components being poorly completed. The QIP was conducted over two, four-month junior doctor rotations with an unforeseen shift to a COVID surge rota in the second rotation. Data was collected several weeks after each intervention to assess its impact.

## Results

**Figure 1:**
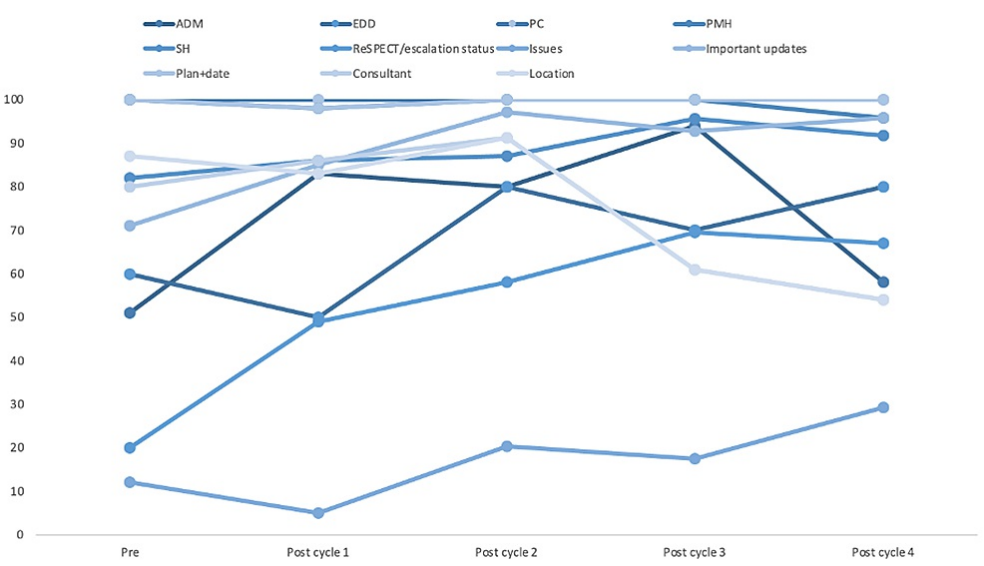
Run chart of percentage completion rate of each component of handover across the four PDSA cycles ADM, admission date; EDD, estimated discharge date; PC, presenting complaint; PMH, past medical history; ReSPECT, escalation status and ceiling of care; SH, social history, PDSA, plan-do-study-act.

The QIP was conducted from August 2020 to February 2021. Data were collected from 710 patient handover records across the four PDSA cycles. Table 2 summarises the percentage completion rates of each component across the cycles. As demonstrated by Figure 1, the percentage completion rate of the ‘Assessment’ section showed the greatest improvement across the four cycles.

**Table 2:** Percentage completion rate of each component of handover on weekend handover lists across four PDSA cycles. ADM, admission date; EDD, estimated discharge date; PC, presenting complaint; PMH, past medical history; ReSPECT, escalation status and ceiling of care; SH, social history; PDSA, plan-do-study-act.

Handover components	Baseline (%)	PDSA1	PDSA2	PDSA3	PDSA4	% change vs baseline
Situation						
ADM	51	83	80	94	58	+13.7%
EDD	60	50	80	70	80	+33.3%
PC	100	100	100	100	100	+0%
Background						
PMH	100	98	100	100	96	-4.0%
SH	82	86	87	96	92	+12.1%
Assessment						
Escalation status (ReSPECT)	20	49	58	70	67	+335%
Issues	12	5	20	17	29	+242%
Important updates	71	85	97	93	96	+35.2%
Recommendations						
Plans + date	100	98	100	100	100	+0%

PDSA 1: Education on handover

To address the issues noted in our baseline audit, we educated foundation doctors on the essential components of a handover in the first PDSA cycle during a mandatory weekly teaching session. This cohort was chosen as it is mainly foundations doctors who are responsible for the day-to-day updating of handovers. They are also more likely to have inconsistent levels of knowledge about handover given the stage of their training. A repeat audit post-intervention demonstrated an improvement in the adherence to the handover proforma, with increased completion rates observed with admission, social history, escalation status and important updates.

PDSA 2: Posters

For a sustained improvement, we focused on increasing awareness of the handover structure more widely by distributing posters of the best practice proforma in doctor’s and MDT offices (Figure 1). Following this intervention, a repeated audit showed continuous or sustained improvement in most key components.

PDSA 3: Careflow champions

In order to have a more direct impact on each ward and involve the wider MDT, who also update handovers, we recruited Careflow Champions to oversee the quality of the handovers and have a more active teaching role within the wards. These were junior doctors who volunteered, and were educated on handover standards through a face-to-face session and a handout was given out. They were also advised on ways to actively involve the MDT in order to improve the handovers. Various wards have different methods of updating handovers, thus Careflow Champions are able to create tailored templates and instigate local change. This stage coincided with the rotation of junior doctors and the emergence of a COVID surge rota. Following recruitment, the data collected showed some improvements but were generally consistent with the results of the previous cycle. The four components that had performed poorly at baseline (ADM, EDD, escalation status and issues) continued to have lower completion rates than the rest of the components.

PDSA 4: Re-education on handover

To compensate for the disruption caused by new rotations, we conducted a further re-education session of the handover at a mandatory foundation teaching. During this cycle, doctors were switched onto the COVID surge rota, leading to redeployment and change in work patterns. Despite these challenges, previously observed improvements were largely retained, although there was a significant decrease in the completion rate of admission dates (94% to 58%) and other components were largely static.

## Discussion

This project has demonstrated that the promotion of a standardised proforma is an effective method in improving the quality of handovers, with serial interventions required to maintain the improvement. The challenges associated with the COVID-19 pandemic have highlighted the importance of a robust handover system, with potential implications for maintaining patient safety and reducing the fragmentation of care.

Electronic handovers have been suggested as a potential solution to improve patient safety [11-13], with the importance in the accessibility of live patient details and management plans being emphasised by the BMA and RCS [5]. Similar studies have explored the impact of electronic handover systems, however, they used subjective measures to review their effectiveness, such as surveys to explore doctors’ views [14-16]. Our QIP by comparison, provides a quantitative analysis, assessing how well e-handovers were updated pre- and post-educational interventions. Our findings are consistent with previous studies which found that utilising e-handovers in conjunction with verbal handovers may improve continuity of patient care [17,18].

The introduction of the COVID surge rota was a significant barrier to sustained improvement during PDSA cycles 3 and 4. Following PDSA cycle 4 in particular, there were reductions in the completion rates of certain components, most significantly observed with the admission dates, and no improvements seen in the majority of components. This may be secondary to the increased turnover of doctors working on specific wards, in conjunction with the aforementioned challenges associated with the pandemic. Ongoing training is vital to ensure new staff are made aware of the key components of handovers. This may be achieved through continuing to include Careflow teaching within the junior doctor induction programme, as well as through departmental inductions. Further efforts are also required to include other members of the MDT, who have an important role in maintaining continuity on the ward when doctors rotate. Owing to the time restraints associated with the general medical admissions cohort, the feasibility of transfer between paper-based medical notes to an electronic handover system remains limited. A transition to fully electronic patient records, which includes handover tools, may overcome this barrier and improve efficiency and sustainability.

One of the key findings of this QIP was the poor completion of the escalation status. The ReSPECT process is an initiative led by the National Resuscitation Council (2021) that promotes shared decision-making when considering the escalation of care and resuscitation status of patients [19]. A ReSPECT form was introduced at Ashford and St. Peter’s Hospitals NHS Foundation Trust in 2018, and all patients are expected to have a completed ReSPECT form on admission [20]. This was identified as one of the key components required in handovers. During our QIP, we have demonstrated a significant improvement in the rate of escalation status updated on the handovers from 20% to 67% by the final PDSA cycle.

The findings of this QIP must be considered within the context of the following limitations. Firstly, although the audited wards were selected randomly to reduce potential bias, this only included three wards at ‘snapshot’ dates post-intervention, limiting the scope of the results. Secondly, teaching sessions were only aimed at junior doctors. Expansion to include other members of the MDT may have yielded further improvements in completion rates.

## Conclusions

Our findings demonstrate an overall improvement in the completion rate of majority of the components of the handover proforma through education. By focusing on standardised components of a safe handover, we were able to obtain a quantitative measure of the standards of handover. Challenges remain in overcoming reduced adherence to the handover proforma following the rotation of junior doctors through different specialties, leading to a loss of institutional knowledge and reduced longevity of the intervention’s effect. Assessing the handovers through the COVID-19 pandemic revealed further difficulties in maintaining quality throughout redeployment and increased work pressures. These challenges highlight the need for a robust handover system such as electronic patient records that incorporate handovers. Nevertheless, this QIP has demonstrated that interventions focusing on educating doctors on handover can lead to increases in the overall quality of handover.
